# Pleiotropic Genes Affecting Carcass Traits in *Bos indicus* (Nellore) Cattle Are Modulators of Growth

**DOI:** 10.1371/journal.pone.0158165

**Published:** 2016-07-13

**Authors:** Anirene G. T. Pereira, Yuri T. Utsunomiya, Marco Milanesi, Rafaela B. P. Torrecilha, Adriana S. Carmo, Haroldo H. R. Neves, Roberto Carvalheiro, Paolo Ajmone-Marsan, Tad S. Sonstegard, Johann Sölkner, Carmen J. Contreras-Castillo, José F. Garcia

**Affiliations:** 1 Departamento de Agroindústria, Alimentos e Nutrição, Escola Superior de Agricultura “Luiz de Queiroz”, USP, Piracicaba, Brazil; 2 Departamento de Medicina Veterinária Preventiva e Reprodução Animal, UNESP–Univ Estadual Paulista, Faculdade de Ciências Agrárias e Veterinárias, Jaboticabal, São Paulo, Brazil; 3 Departamento de Apoio, Produção e Saúde Animal, UNESP—Univ Estadual Paulista, Faculdade de Medicina Veterinária de Araçatuba, Araçatuba, São Paulo, Brazil; 4 International Atomic Energy Agency (IAEA) Collaborating Centre on Animal Genomics and Bioinformatics, Araçatuba, São Paulo, Brazil; 5 GenSys Consultores Associados, Porto Alegre, Brazil; 6 Departamento de Zootecnia, UNESP—Univ. Estadual Paulista, Faculdade de Ciências Agrárias e Veterinárias, Jaboticabal, São Paulo, Brazil; 7 Università Cattolica del Sacro Cuore, Piacenza, Italy; 8 Recombinetics, Inc., St Paul, MN, United States of America; 9 BOKU—University of Natural Resources and Life Sciences, Department of Sustainable Agricultural Systems, Division of Livestock Sciences, Vienna, Austria; Faculty of Animal Sciences and Food Engineering, University of São Paulo, BRAZIL

## Abstract

Two complementary methods, namely Multi-Trait Meta-Analysis and Versatile Gene-Based Test for Genome-wide Association Studies (VEGAS), were used to identify putative pleiotropic genes affecting carcass traits in *Bos indicus* (Nellore) cattle. The genotypic data comprised over 777,000 single-nucleotide polymorphism markers scored in 995 bulls, and the phenotypic data included deregressed breeding values (dEBV) for weight measurements at birth, weaning and yearling, as well visual scores taken at weaning and yearling for carcass finishing precocity, conformation and muscling. Both analyses pointed to the pleomorphic adenoma gene 1 (*PLAG1*) as a major pleiotropic gene. VEGAS analysis revealed 224 additional candidates. From these, 57 participated, together with *PLAG1*, in a network involved in the modulation of the function and expression of *IGF1* (insulin like growth factor 1), *IGF2* (insulin like growth factor 2), *GH1* (growth hormone 1), *IGF1R* (insulin like growth factor 1 receptor) and *GHR* (growth hormone receptor), suggesting that those pleiotropic genes operate as satellite regulators of the growth pathway.

## Introduction

Carcass yield plays a major economic role in beef cattle, as the carcass retail price is essentially determined by its weight. As differences in carcass yield between steers are partially heritable, selection and breeding are determinant operations in the beef cattle sector [1]. However, direct carcass measurements are challenging as phenotype collection depends on animal slaughter. Therefore, the use of surrogate phenotypes such as body weight measurements and visual carcass evaluation in live animals is imperative for improving carcass yield [2].

Weight measurements and visual scores of conformation, carcass finishing precocity and muscling (CPM) have been routinely employed in selection to improve carcass yield in Brazilian Nellore (*Bos indicus*) cattle. These traits are inexpensive and effortless to measure, and present moderate heritability in the breed [3]. However, these different traits supposedly have distinct genetic architecture, and determining the extent of their genetic correlations, as well as identifying genes affecting multiple traits simultaneously (i.e. pleiotropic genes) would be beneficial to improve strategies for genetic selection.

Bolormaa et al. [4] have recently described a method for mapping pleiotropic variants affecting traits of interest in beef cattle. The procedure consists in performing genome-wide association (GWA) scans for each trait separately, and then summarizing the effects of each genetic marker across traits with a meta-analytical approach. Additionally, other recently developed methods have aimed at increasing power and interpretability of association studies by combining single-marker results within functional elements (e.g. genes) or user-specified chromosomal windows [5,6]. Combining these two approaches may be useful in the search for putative pleiotropic genes affecting traits of interest in animal breeding. Here, we attempted to apply these methods to a sample of 995 Nellore bulls genotyped for over 777,000 single-nucleotide polymorphism (SNP) markers, for which deregressed estimated breeding values (dEBV) were available for nine weight and CPM traits. More specifically, we aimed at identifying major pleiotropic genes underlying variation in traits that are predictive of carcass yield in *B*. *indicus* cattle.

## Materials and Methods

### Ethical statement

This study was exempt from the local ethical committee evaluation as DNA samples used for genotyping were obtained from industrialized semen straws.

### Genotypes

A total of 995 Nellore bulls were genotyped with the Illumina® BovineHD Genotyping BeadChip assay, according to the manufacturer's protocol. The panel included 777,962 SNPs annotated in the UMD v3.1 bovine genome assembly. These bulls are part of the genomic selection reference population from a commercial breeding program, namely DeltaGen (http://www.deltagen.com.br/nelore.php). Data filtering was performed with PLINK v1.9 [7,8]. All genotyped samples had call rate greater than 90%. Only autosomal markers presenting a minimum call rate of 95% and a minor allele frequency of at least 2% were analyzed.

### Estimated breeding values and variance components

Estimated breeding values (EBVs) for birth weight (BW), weaning gain (WG), conformation at weaning (CW), carcass finishing precocity at weaning (PW), muscling at weaning (MW), post-weaning gain (PG), conformation at yearling (CY), carcass finishing precocity at yearling (PY), and muscling at yearling (MY) were obtained from routine genetic evaluations. The single-trait animal models used to generate the EBVs were corrected for environmental and maternal effects, and included records from 1,278,057 animals born between 1985 and 2012, and raised in 315 grazing-based Brazilian herds. The variance ratios required to solve the mixed model equations were computed based on restricted maximum likelihood estimates of the variance components. The heritabilities obtained for BW, WG, CW, PW, MW, PG, CY, PY and MY were 0.37, 0.26, 0.25, 0.25, 0.26, 0.33, 0.31, 0.31 and 0.30, respectively. Prior to the association analysis, EBVs were deregressed following [9], and only the bulls presenting deregressed EBVs with a minimum accuracy (based on prediction error variance) of 0.50 were analyzed.

Records for WG and PG were based on the weight gain from birth to weaning (adjusted for a period of 205 days) and from weaning to yearling (adjusted for a period of 550 days), respectively. Records for conformation, carcass finishing precocity and muscling were taken at weaning and yearling based on visual score evaluations relative to the animals of the same management group. Scores were assigned in a discrete ordered scale ranging from 1 to 5. The model used for BW included the fixed effects of contemporary group (defined as animals from the same herd, born in the same year and season, and belonging to the same birth management group) and age of dam at calving, as well as random maternal effects (maternal additive genetic effect and maternal permanent environmental effect). The model used for weaning traits included fixed effects of contemporary group (concatenation of BW contemporary group and herd-management group at weaning), Julian birth date within birth season, age at phenotype recording and age of dam at calving, in addition to the maternal effects described for BW. Post-weaning gain and the remaining yearling traits were corrected for the fixed effects of contemporary group (concatenation of WG contemporary group and herd-management group at yearling), age at phenotype recording and age of dam at calving.

### Regression model

Prior to testing SNPs for association, the same single-trait regression model was applied across traits:

$$y=1_{n}\mu +g+e$$

where **y** is the *n* x 1 vector of dEBVs, **1**_*n*_ is a *n* x 1 vector of 1s, μ is the overall mean, **g** is the *n* x 1 vector of random polygenic effects, and **e** is the *n* x 1 vector of random residual effects. Vector **g** was assumed a linear combination of additive marker effects:

g=Xb

where **X** is a *n* x *m* matrix of allele dosages at *m* markers (coded as 0, 1 or 2 copies of the minor allele), and **b** is a *m* x 1 vector of random additive marker effects. Vector **g** was assumed N(0, **G**), where **G** = **Kσ**_g_^2^, **K** is the *n* x *n* matrix of additive relationships between individuals, and **σ**_g_^2^ is the variance due to genome-wide markers. Vector **e** was assumed N(0, **R**), where **R** = **Wσ**_e_^2^, **W** is a diagonal matrix of weights accounting for heterogeneity of variance in dEBVs, and **σ**_e_^2^ is the residual variance. The resulting variance-covariance matrix of the model was **V** = **G** + **R**. Notice that this is essentially the polygenic [10,11] or the Genomic Best Linear Unbiased Predictor (GBLUP) model [12] corrected for heteroscedastic residuals. Estimates of genetic parameters for each trait were obtained using the hglm v2.1–0 package in R v3.2.1 [13].

### Choice of residual weights

Following Garrick et al.[9], the diagonal elements of **W** were computed as **w** = λ^-1^(**d** + *c*), where λ = (1-*h*^*2*^)/*h*^*2*^, *h*^*2*^ is the heritability of the trait before deregression, **d** = (1 –**r**^**2**^)/**r**^**2**^, **r**^**2**^ is the squared vector of accuracies (i.e., reliabilities) of the pseudo-phenotypes, and *c* is a parameter taking values between 0 and 1 controlling the relative contribution of pseudo-phenotypes on the basis of their reliabilities. As can be seen in the formula, the weights **w** used here are linearly related to the weights **d** used by Neves et al. [14], except that they were scaled by the variance ratio λ and added by a constant *c*. In order to achieve a balanced contrast between dEBVs with high and low accuracy, we adopted *c* = 0.5.

### GWA analysis

Conceptually, associations are tested by contrasting the null polygenic model against alternative models that include the fixed effect of one candidate marker at a time [11,15,16]. However, this contrast is redundant since the candidate marker was also included as a random effect in the null model through **g** = **Xb**. This introduces a bias known as 'proximal contamination' [17], which can substantially reduce the power of the tests. In order to avoid it, we used the leave-one-chromosome-out approach described by Yang et al. [18]. Briefly, the method consists in partitioning the genome-wide scan procedure per chromosome. For each chromosome *j*, we fit a modified null model where matrix **K** is built excluding all markers on *j*, guaranteeing that the null model does not contain the marker being tested or any other marker in linkage disequilibrium (LD) with it. Then, each marker on chromosome *j* is contrasted against the modified null model by using the test statistic *t* = *b*/*SE(b)*, where *b* = (**x'V**^**-1**^**x**)^-1^ **x'V**^**-1**^y* for y* = **y—1**μ and *SE(b)* is the square root of *VAR*(b) = (**x'V**^**-1**^**x**)^-1^. In this way, *t* is conditional on the random effects of genome-wide markers, such that the model preserves power while correcting for relatedness and population substructure. Additionally, by incorporating matrix **W** on **V**, we accounted for heterogeneity of variance in dEBVs while estimating *b* and *SE(b)*. Estimates for *b* and *SE(b)* were obtained by providing **V** and y* to the mmscore function in GenABEL v1.8–0 [19]. In summary, our single-trait GWA analysis was almost identical to the leave-one-chromosome-out (i.e. mlma-loco) procedure [18] in GCTA [20], except that our model accounted for heterogeneity in residual variance.

### Detection of pleiotropic genes

In order to identify pleiotropic genes affecting CPM and body weight traits, we combined two distinct but complementary strategies. The Multi-Trait Meta-Analysis method described by Bolormaa et al. [4] was used to summarize single-marker statistics across all studied traits and detect major pleiotropic genes. Additionally, the Versatile Gene-Based Test for Genome-wide Association Studies (VEGAS) method [6] was applied to the single-trait associations to perform gene-set based analyses, and genes appearing in the significant list of at least four of the nine traits (i.e., approximately half of the traits) evaluated were considered as candidate pleiotropic genes. VEGAS and Multi-Trait Meta-Analysis were implemented in R v3.2.1 and are described below.

#### Multi-trait meta-analysis

For each SNP, consider **t** as the *q* x 1 vector of signed *t*-values across *q* traits, and **C** the *q* x *q* matrix of *t*-values correlations across genome-wide markers. The test statistic **t'C**^**-1**^**t** is distributed as χ^2^ with *q* degrees of freedom (df) under the null hypothesis of no pleiotropic effect. One standing issue in this implementation is that assuming **C**_*i*,*i*_ = 1 when some traits present higher average correlations than others may cause highly significant composite scores even when single-trait analyses collectively present poor evidence of association. This issue was corrected here by adding the average correlation of each trait to their respective diagonal elements. The expected proportion of false discoveries among the markers declared significant was computed as *f* = α*m*/*s*, where *m* is the number of tests, α is the significance level threshold, and *s* is the number of tests with *p* < α. In order to select a value of α resulting in a false discovery rate lower than 5%, we applied the procedure described by [21]. Briefly, we manipulated the expression above to obtain α = *fs*/*m* and defined *s* as the largest *p*-value rank position *i* satisfying *p*_*i*_ ≤ *f*_*i*_/*m* for *f* = 0.05.

#### VEGAS

For each trait and for each gene, the joint VEGAS test was computed as the sum of squared *t*-values across markers. The distribution of the VEGAS test under the null hypothesis of no association is unknown but can be approximated using Monte Carlo simulations. The simulation procedure is carried out as follows: random draws from the null distribution are generated as *x*^2^ = *z*_1_^2^ + *z*_*2*_^2^ + … + *z*_*m*_^2^, where *z*_*m*_^2^ is one element of a *m* x 1 random vector **z** sampled from a multivariate normal distribution with mean **0**_*q*_ and covariance **D**, encoded by the matrix of signed genotypic correlations among the *m* markers within the gene. The probability of observing a VEGAS statistic value as extreme as the one obtained if the null hypothesis is true is then computed as the number of times the simulated *x*^2^ values were greater than or equal to the observed VEGAS statistic, divided by the total number of simulations. The number of simulations required to approximate the null distribution is chosen adaptively: let ***x***^**2**^ be the vector of random draws from the null distribution. At every iteration, if the *p*-value is lower than the inverse of the current number of simulations *k*, *k*—length(***x***^**2**^) extra samples are obtained, the new *p*-value is computed, and the new number of simulations is set to 10^log10(*k*)+1^. We initialized the iterations using *k* = 1,000. In this way, the algorithm dynamically re-calibrated low *p*-values based on a sample precision of 10^3^, 10^4^, 10^5^ and 10^6^ simulations. Due to computational limitations, the process was interrupted at 10^6^ simulations, and probabilities were bounded to *p* < 10^−6^. Genes sheltering no SNPs had their *p*-values set to 1. In order to guarantee that **D** was positive definite, we used the maximum set of markers for which all pairwise squared correlations were lower than or equal to 0.5. In order to capture information from intergenic markers in LD with unobserved variants lying within genes and their regulatory regions, we expanded gene boundaries in the UMDv3.1 assembly to ± 100 kb of the 5' and 3' UTRs. Finally, given the VEGAS test is highly conservative, the gene list for each trait comprised all genes with *p* < 0.01. Based on the moderate genetic correlations among the nine traits studied here (see the Results and Discussion section), putative pleiotropic genes were defined as those appearing in the gene list of four or more traits, corresponding to genes potentially associated with at least half of the studied traits.

### Functional analysis

Interactions between protein-coding genes were predicted using the STRING database [22]. Additionally, networks were graphed with gephi 0.8.2 (available at: http://gephi.github.io/).

## Results and Discussion

### Data filtering

A total of 516,740 SNPs and 995 individuals passed the filtering criteria and were retained in the dataset. After data filtering, the mean and median gap between any pair of consecutive markers on the same chromosome were approximately 5.26 kb and 3.15 kb, respectively. The resulting genotyping rate across markers and samples was approximately 0.99. The number of bulls with dEBV accuracy higher than 0.5 for BW, WG, CW, PW, MW, PG, CY, PY and MY were 837, 915, 875, 876, 875, 880, 844, 844 and 844, respectively.

### Evidence of pleiotropic effect from additive genetic correlations

As dEBVs encapsulate individual additive genetic values and association *t*-values represent marked additive genetic effects, we considered both dEBVs and *t*-values correlations between traits as proxies for the additive genetic correlations between traits. On average (**Table 1**), dEBVs were moderately correlated across traits (r = 0.442), and strikingly similar results were found for *t*-values across traits (r = 0.423). An exception was BW, which was only mildly correlated with WG and moderately correlated with conformation traits. These noteworthy genetic correlations suggest that pleiotropic loci may contribute to the genetic variance of these traits.

**Table 1:** Deregressed estimated breeding values (dEBV) and genome-wide SNP effects correlations (inside brackets) for weight and carcass traits in *Bos indicus* (Nellore) bulls.

Trait	BW	WG	PG	CW	PW	MW	CY	PY	MY
BW	1.000 (1.000)	0.140 (0.202)	0.079 (0.062)	0.277 (0.321)	-0.059 (-0.063)	-0.056 (-0.045)	0.217 (0.272)	-0.051 (-0.078)	-0.052 (-0.070)
WG	0.140 (0.202)	1.000 (1.000)	0.501 (0.490)	0.776 (0.790)	0.493 (0.473)	0.507 (0.483)	0.734 (0.751)	0.435 (0.410)	0.430 (0.406)
PG	0.079 (0.062)	0.501 (0.490)	1.000 (1.000)	0.351 (0.360)	0.291(0.238)	0.234 (0.184)	0.571 (0.604)	0.456 (0.441)	0.429 (0.425)
CW	0.277 (0.321)	0.776 (0.800)	0.351 (0.360)	1.000 (1.000)	0.381 (0.325)	0.412 (0.359)	0.884 (0.877)	0.329 (0.265)	0.335 (0.274)
PW	-0.059 (-0.063)	0.493 (0.473)	0.291 (0.238)	0.381 (0.325)	1.000 (1.000)	0.884 (0.886)	0.393 (0.308)	0.917 (0.890)	0.833 (0.802)
MW	-0.056 (-0.044)	0.507 (0.483)	0.234 (0.185)	0.412 (0.359)	0.884 (0.886)	1.000 (1.000)	0.382 (0.303)	0.798 (0.770)	0.888 (0.855)
CY	0.217 (0.272)	0.734 (0.751)	0.571 (0.604)	0.884 (0.877)	0.393 (0.308)	0.382 (0.303)	1.000 (1.000)	0.451 (0.384)	0.445 (0.383)
PY	-0.051 (-0.078)	0.435 (0.410)	0.456 (0.441)	0.329 (0.265)	0.917 (0.890)	0.798 (0.770)	0.451 (0.384)	1.000 (1.000)	0.885 (0.887)
MY	-0.052 (-0.070)	0.430 (0.407)	0.429 (0.425)	0.335 (0.274)	0.833 (0.802)	0.888 (0.853)	0.445 (0.383)	0.885 (0.887)	1.000 (1.000)

BW = birth weight; WG = weaning gain; PG = post-weaning gain; CW = conformation at weaning; PW = carcass finishing precocity at weaning; MW = muscling at weaning; CY = conformation at yearling; PY = carcass finishing precocity at yearling; MY = muscling at yearling.

### Major pleiotropic effects map to the *PLAG1* region

**Fig 1:**
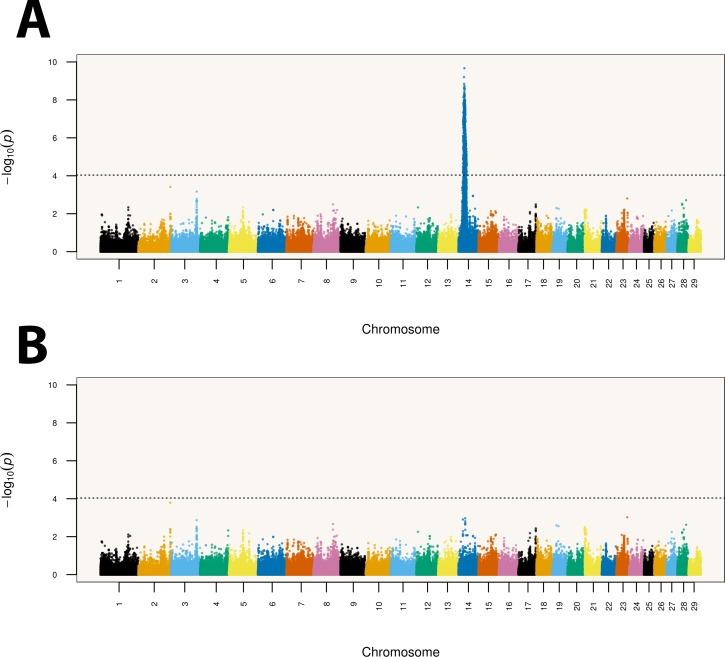
Genome-wide Multi-Trait Meta-Analysis for loci affecting carcass traits in *Bos indicus* (Nellore) cattle. The dashed horizontal line represents the significance threshold (*p* < 9.20 x 10^−5^). Results are shown before (A) and after (B) the removal of the effect of marker rs136543212 (probe ID BovineHD1400007373).

After combining the results across the nine traits with the Multi-Trait Meta-Analysis method, a total of 983 markers were declared significant at an empirical threshold of *p* < 9.20 x 10^−5^ (**Fig 1A**), resulting in a false discovery rate of approximately 5%. A single large, dominant signal mapping to chromosome 14:19.46–34.92 Mb was identified. The leading SNP, namely rs136543212 (probe BovineHD1400007373), mapped to position 25,502,915, in the vicinity of the well-known *PLAG1* (pleomorphic adenoma gene 1) chromosome domain.

This chromosomal segment has been implicated as a highly pleiotropic locus underlying genetic differences in growth, weight and reproductive traits in cattle [4,23–29]. The functional candidacy of *PLAG1* is well supported by its regulation of the expression of insulin-like growth factors (IGF) [30]. These factors are major mediators of the growth pathway and the hypothalamic-pituitary-gonadal axis [31], and serum concentrations of IGF1 in cattle have been shown to be highly heritable, negatively correlated with weight and carcass traits [32,33] and primarily determined by variants in the *PLAG1* chromosomal region [29]. Nevertheless, the identities of the causal variant and the affected genes are still to be unraveled, since other genes in the vicinity of *PLAG1* are also plausible functional candidates, notably *MOS* [23], *CHCHD7* [23,34], *XKR4* [35–37] and *PENK* [38–40]. The signal detected here suggested that the underlying causal variant has negative effects on carcass finishing precocity/muscling traits and positive effects on weight/conformation traits, consistent with a previous report of a candidate causal variant [23] of *Bos taurus* origin (the C allele at SNP rs109231213, position 14:25003338) associated with decreased IGF1 serum concentrations and precocity, as well as with increased height and weight [28].

**Fig 2:**
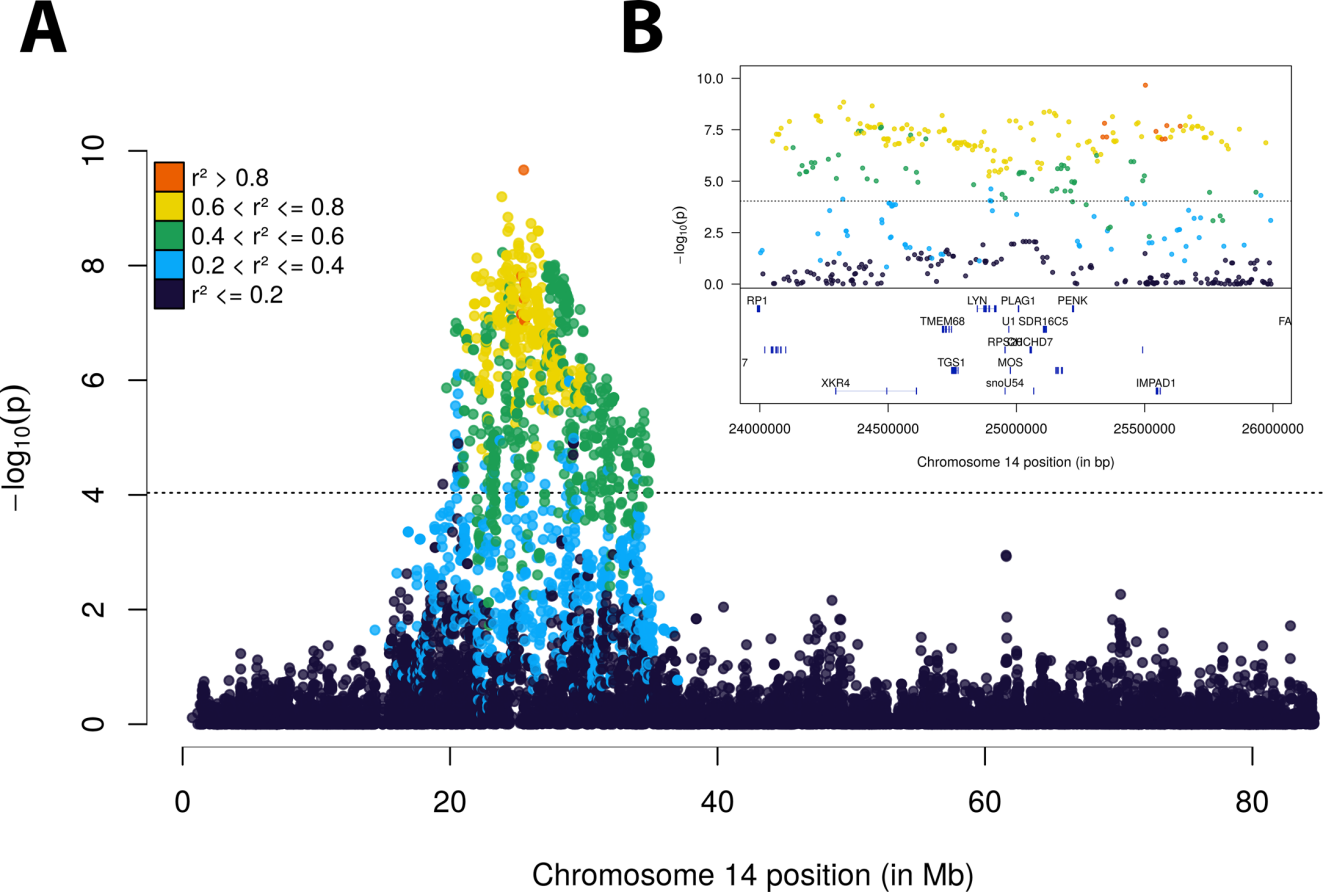
Association plot of chromosome 14 region. LD (r^2^) with the top scoring marker rs136543212 (probe ID BovineHD1400007373) is represented according to the indicated color scale. The dashed horizontal line represents the significance threshold (*p* < 9.20 x 10^−5^). The chromosome-wise plot (A) and the regional plot around *PLAG1* (B) reveal that the association is driven by a single large LD block.

To determine if this large segment comprised a single signal driven by a large LD block or it construed a mixture of signals, we re-analyzed our data using the same GWA model conditional on the fixed effect of the top scoring SNP. Correction for the effect of rs136543212 alone was able to remove most of the signal (**Fig 1B**), which suggests that this large segment is a single LD block. In fact, all significant markers were in moderate to high LD with the top scoring marker (**Fig 2**). However, as correcting for the leading SNP was not sufficient to completely eliminate the signal, it is hard to distinguish between the presence of more than one causal nucleotide within the LD block and residual effects captured by the remaining markers due to imperfect tagging of rs136543212.

### Detection and interactions of additional candidate pleiotropic genes

We carried out the VEGAS analysis using single-trait GWA results. The top scoring gene appearing in the gene list of eight of the nine traits investigated was *PLAG1*, consistent with the findings of the Multi-Trait Meta-Analysis approach. Our analysis showed that the VEGAS and the Multi-Trait Meta-Analysis approaches are complementary, and that they can be used jointly to maximize the discovery of pleiotropic genes. As the *PLAG1* signal comprised a large LD block, we carried out further search for pleiotropic genes omitting results from chromosome 14, except for *PLAG1* itself. Additionally, we also omitted results from 25 olfactory receptor genes and a cluster of 32 histone genes, both mapping to the vicinity of other functional candidate genes. After applying these filters, we obtained a list of 224 candidate pleiotropic genes. These included 176 protein-coding genes, 12 pseudo-genes, 12 snoRNA, 11 snRNA, 11 miRNA and two misc_RNA. We then focused our functional annotation on protein-coding genes.

Besides *PLAG1*, we found a series of growth-related genes, including growth differentiation factors 2 (*GDF2*) [41], 10 (*GDF10*) [42] and 11 (*GDF11*) [43], growth arrest-specific 2 like 3 (*GAS2L3*) [44], fibroblast growth factor 22 (*FGF22*) [45], PH domain and leucine rich repeat protein phosphatase 1 (*PHLPP1*) [46], signal transducer and activator of transcription 2 (*STAT2*) [47], SMAD family member 4 (*SMAD4*) [48], and insulin-like growth factor binding protein 5 (*IGFBP5*) [49]. Genes involved in muscle development and function were also found, including methylmalonyl CoA mutase (*MUT*) [50], troponin T type 1 (*TNNT1*) [51], troponin I type 3 (*TNNI3*) [52], and sarcoglycan delta (*SGCD*, also known as 35kDa dystrophin-associated glycoprotein) [53].

**Fig 3:**
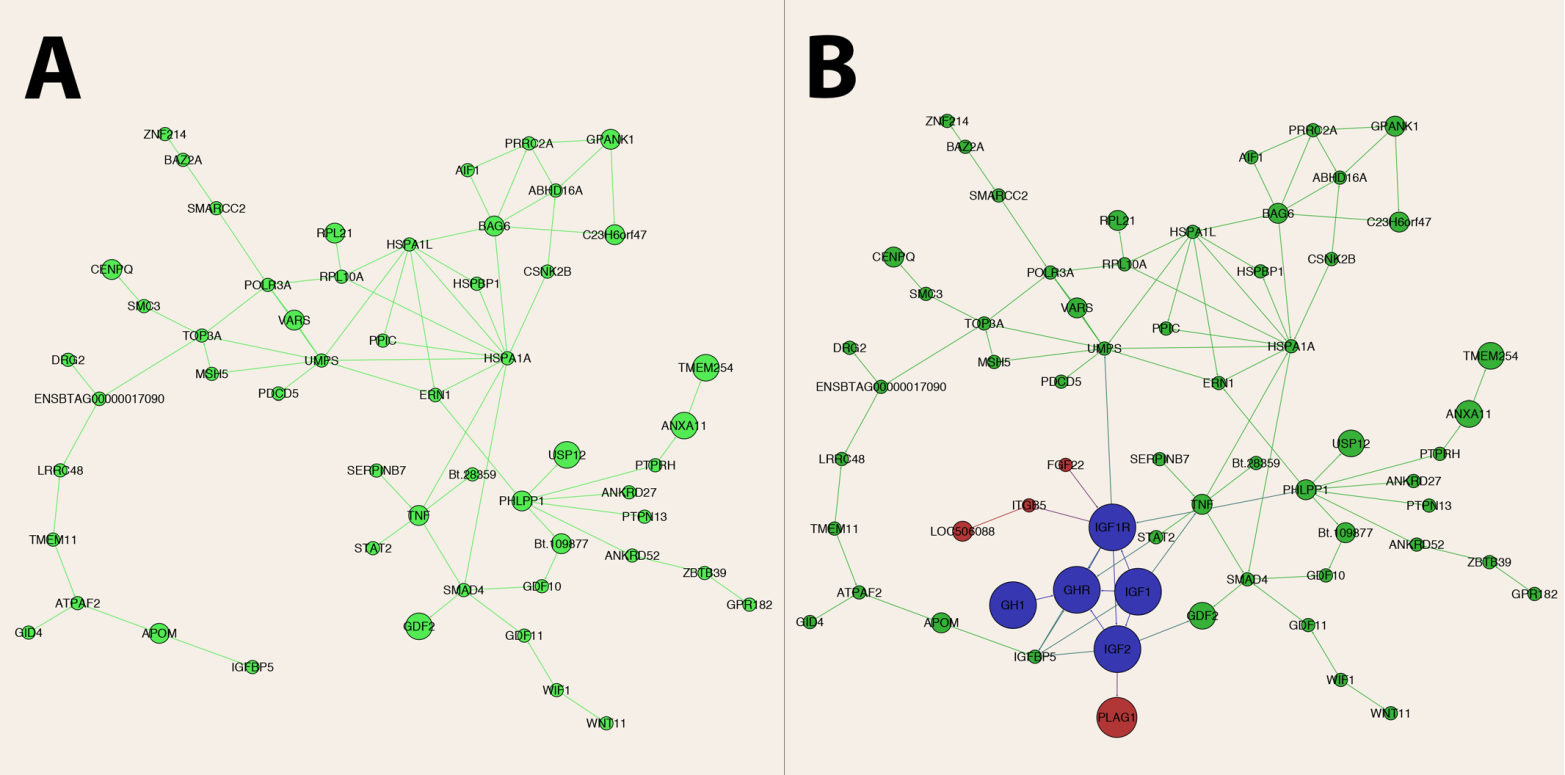
Network of candidate pleiotropic genes for carcass traits in *Bos indicus* (Nellore) cattle. The network was built from known protein-protein interactions (edges) between gene products (nodes). The size of the node is proportional to the number of traits the gene is associated with. In A, the network is portrayed according to the list of genes obtained from the VEGAS analyses. In B, after the inclusion of five essential genes (in blue) form the growth pathway, the network presented itself as a satellite, and four more genes (in red) could be incorporated, including the major pleotropic gene *PLAG1*.

We then used the STRING database to annotate protein-protein interactions among the candidate genes. From the initial list of 176 genes, 82 presented connections. From these, 54 genes were involved in a single network (**Fig 3A**). The remaining 28 genes formed smaller networks ranging from two to six genes (**S1 Data**). Interestingly, *PLAG1* was not present in any network, in spite of being the major pleiotropic gene in our study.

Although key genes involved in growth, such as *IGF1* (insulin like growth factor 1), *IGF2* (insulin like growth factor 2), *GH1* (growth hormone 1), *IGF1R* (insulin like growth factor 1 receptor) and *GHR* (growth hormone receptor), were not found to shelter pleiotropic variants affecting carcass traits in the present study, we decided to add them to the list to reveal their interactions with the pleiotropic genes found here (**Fig 3B**). The main motivation for this was the known function of *PLAG1*: by fine-tuning the expression of the IGF family, the predicted downstream consequence of mutations in *PLAG1* is direct interference in the growth pathway. This could also be the case for other pleiotropic genes identified here. Another argument to support this strategy was that mutations directly occurring in the growth pathway are likely to produce extreme phenotypes, such that common variation in carcass size is expected to be explained by variation in satellite genes modulating that pathway. For instance, mutations in *IGF1*, *IGF1R* and *GHR* have been implicated in extreme body size reduction across dog breeds [54], as opposed to common variation in human height being explained by a large number of variants in genes outside the growth pathway [55].

Surprisingly, when *IGF1*, *IGF2*, *GH1*, *IGF1R* and *GHR* were added to the gene list, the pleiotropic genes formed a satellite network surrounding a central hub comprising the growth pathway. Additionally, *FGF22*, *ITGB5*, *LOC506088* and *PLAG1* were incorporated to the network when the genes mentioned above were included. This finding supported our hypothesis that the pleiotropic genes detected here are modulators of the growth pathway. It also opens the question whether genetic correlations among the weight and CPM traits encapsulate information about the growth pathway, in the sense that each one of these traits may be serving as a partial surrogate for the same underlying intermediate trait (i.e. growth). In this scenario, one would expect to find similar results in a direct GWA analysis on the intermediate phenotype.

## Conclusions

We identified genes associated with multiple carcass traits in *Bos indicus* (Nellore) cattle. These pleiotropic genes formed a network modulating *IGF1*, *IGF1R*, *IGF2*, *GH1* and *GHR*, which are well known major actors of the growth pathway. This finding suggests that common variation in carcass traits is not likely to be explained by mutations in essential genes controlling growth. Instead, the variation may lie in accessory genes that regulate the function and expression of essential genes. Among these accessory genes, *PLAG1* seems to be the most influential. Moreover, we did not rule out the possibility that, at some extent, the genetic correlations among the nine traits studied here represent the indirect, partial measurement of a single underlying growth trait. In this case, the pleiotropic genes identified here may simply represent genes affecting the intermediate phenotypes. The future characterization of causal variants in these genes may contribute to improved prediction of carcass yield and more informed mating decisions in *B*. *indicus* cattle.

## Supporting Information

S1 Data GWA data including detailed information of the single-trait and multi-trait analyses, as well as gene description results for the VEGAS and STRING analyses. (GZ)

## References

[1] Garrick DJ. The nature, scope and impact of genomic prediction in beef cattle in the United States. Genet Sel Evol. BioMed Central Ltd; 2011;43: 17 10.1186/1297-9686-43-17 21569623 PMC3107171

[2] Cancian PH, Gomes R da C, Manicardi FR, Ianni AC, Bonin M de N, Leme PR, et al Correlations of visual scores, carcass traits, feed efficiency and retail product yield in Nellore cattle. Sci Agric. Scientia Agricola; 2014;71: 17–22. 10.1590/S0103-90162014000100002

[3] Boligon AA, Mercadante MEZ, Albuquerque LG. Genetic associations of conformation, finishing precocity and muscling visual scores with mature weight in Nelore cattle. Livest Sci. 2011;135: 238–243. 10.1016/j.livsci.2010.07.011

[4] Bolormaa S, Pryce JE, Reverter A, Zhang Y, Barendse W, Kemper K, et al A multi-trait, meta-analysis for detecting pleiotropic polymorphisms for stature, fatness and reproduction in beef cattle. PLoS Genet. 2014;10: e1004198 10.1371/journal.pgen.1004198 24675618 PMC3967938

[5] Capomaccio S, Milanesi M, Bomba L, Vajana E, Ajmone-Marsan P. MUGBAS: a species free gene-based programme suite for post-GWAS analysis. Bioinformatics. 2015;31: 2380–1. 10.1093/bioinformatics/btv144 25765345

[6] Liu JZ, McRae AF, Nyholt DR, Medland SE, Wray NR, Brown KM, et al A versatile gene-based test for genome-wide association studies. Am J Hum Genet. 2010;87: 139–45. 10.1016/j.ajhg.2010.06.009 20598278 PMC2896770

[7] Chang CC, Chow CC, Tellier LC, Vattikuti S, Purcell SM, Lee JJ. Second-generation PLINK: rising to the challenge of larger and richer datasets. Gigascience. 2015;4: 7 10.1186/s13742-015-0047-8 25722852 PMC4342193

[8] Purcell S, Neale B, Todd-Brown K, Thomas L, Ferreira MAR, Bender D, et al PLINK: a tool set for whole-genome association and population-based linkage analyses. Am J Hum Genet. 2007;81: 559–75. 10.1086/519795 17701901 PMC1950838

[9] Garrick DJ, Taylor JF, Fernando RL. Deregressing estimated breeding values and weighting information for genomic regression analyses. Genet Sel Evol. 2009;41: 55 10.1186/1297-9686-41-55 20043827 PMC2817680

[10] Amin N, van Duijn CM, Aulchenko YS. A genomic background based method for association analysis in related individuals. PLoS One. 2007;2: e1274 10.1371/journal.pone.0001274 18060068 PMC2093991

[11] Chen W-M, Abecasis GR. Family-based association tests for genomewide association scans. Am J Hum Genet. 2007;81: 913–26. 10.1086/521580 17924335 PMC2265659

[12] Taylor JF. Implementation and accuracy of genomic selection. Aquaculture. 2014;420–421: S8–S14. 10.1016/j.aquaculture.2013.02.017

[13] Rönnegrard L, Shen X, Alam M. hglm: A Package for Fitting Hierarchical Generalized Linear Models. 2010;2. Available: https://www.researchgate.net/publication/210052870_hglm_A_Package_for_Fitting_Hierarchical_Generalized_Linear_Models

[14] Neves HHR, Carvalheiro R, O’Brien AMP, Utsunomiya YT, do Carmo AS, Schenkel FS, et al Accuracy of genomic predictions in Bos indicus (Nellore) cattle. Genet Sel Evol. 2014;46: 17 10.1186/1297-9686-46-17 24575732 PMC4014866

[15] Kang HM, Sul JH, Service SK, Zaitlen NA, Kong S-Y, Freimer NB, et al Variance component model to account for sample structure in genome-wide association studies. Nat Genet. Nature Publishing Group; 2010;42: 348–54. 10.1038/ng.548 20208533 PMC3092069

[16] Lippert C, Listgarten J, Liu Y, Kadie CM, Davidson RI, Heckerman D. FaST linear mixed models for genome-wide association studies. Nat Methods. Nature Publishing Group, a division of Macmillan Publishers Limited. All Rights Reserved.; 2011;8: 833–5. 10.1038/nmeth.1681 21892150

[17] Listgarten J, Lippert C, Kadie CM, Davidson RI, Eskin E, Heckerman D. Improved linear mixed models for genome-wide association studies. Nat Methods. Nature Publishing Group, a division of Macmillan Publishers Limited. All Rights Reserved.; 2012;9: 525–6. 10.1038/nmeth.2037 22669648 PMC3597090

[18] Yang J, Zaitlen NA, Goddard ME, Visscher PM, Price AL. Advantages and pitfalls in the application of mixed-model association methods. Nat Genet. Nature Publishing Group, a division of Macmillan Publishers Limited. All Rights Reserved.; 2014;46: 100–6. 10.1038/ng.2876 24473328 PMC3989144

[19] Aulchenko YS, Ripke S, Isaacs A, van Duijn CM. GenABEL: an R library for genome-wide association analysis. Bioinformatics. 2007;23: 1294–6. 10.1093/bioinformatics/btm108 17384015

[20] Yang J, Lee SH, Goddard ME, Visscher PM. GCTA: a tool for genome-wide complex trait analysis. Am J Hum Genet. 2011;88: 76–82. 10.1016/j.ajhg.2010.11.011 21167468 PMC3014363

[21] Benjamini Y, Hochberg Y. Controlling The False Discovery Rate—A Practical And Powerful Approach To Multiple Testing. J R Stat Soc Ser B Methodol. 1995;57: 289–300. 10.2307/2346101

[22] Szklarczyk D, Franceschini A, Wyder S, Forslund K, Heller D, Huerta-Cepas J, et al STRING v10: protein-protein interaction networks, integrated over the tree of life. Nucleic Acids Res. 2015;43: D447–52. 10.1093/nar/gku1003 25352553 PMC4383874

[23] Karim L, Takeda H, Lin L, Druet T, Arias JAC, Baurain D, et al Variants modulating the expression of a chromosome domain encompassing PLAG1 influence bovine stature. Nat Genet. 2011;43: 405–13. 10.1038/ng.814 21516082

[24] Littlejohn M, Grala T, Sanders K. Genetic variation in PLAG1 associates with early life body weight and peripubertal weight and growth in Bos taurus. … Genet. 2012; Available: http://onlinelibrary.wiley.com/doi/10.1111/j.1365-2052.2011.02293.x/full 10.1111/j.1365-2052.2011.02293.x 22497486

[25] Utsunomiya YT, Carmo AS, Neves HHR, Carvalheiro R, Matos MC, Zavarez LB, et al Genome-wide mapping of loci explaining variance in scrotal circumference in Nellore cattle. PLoS One. Public Library of Science; 2014;9: e88561 10.1371/journal.pone.0088561 24558400 PMC3928245

[26] Utsunomiya YT, do Carmo AS, Carvalheiro R, Neves HHR, Matos MC, Zavarez LB, et al Genome-wide association study for birth weight in Nellore cattle points to previously described orthologous genes affecting human and bovine height. BMC Genet. 2013;14: 52 10.1186/1471-2156-14-52 23758625 PMC3683327

[27] Fortes MRS, Lehnert SA, Bolormaa S, Reich C, Fordyce G, Corbet NJ, et al Finding genes for economically important traits: Brahman cattle puberty. Anim Prod Sci. CSIRO PUBLISHING; 2012;52: 143 10.1071/AN11165

[28] Fortes M, Kemper K, Sasazaki S. Evidence for pleiotropism and recent selection in the PLAG1 region in Australian Beef cattle. Anim …. 2013; Available: http://onlinelibrary.wiley.com/doi/10.1111/age.12075/full 10.1111/age.12075 23909810

[29] Fortes MRS, Reverter A, Kelly M, McCulloch R, Lehnert SA. Genome-wide association study for inhibin, luteinizing hormone, insulin-like growth factor 1, testicular size and semen traits in bovine species. Andrology. 2013;1: 644–50. 10.1111/j.2047-2927.2013.00101.x 23785023

[30] Voz ML, Agten NS, Van de Ven WJ, Kas K. PLAG1, the main translocation target in pleomorphic adenoma of the salivary glands, is a positive regulator of IGF-II. Cancer Res. 2000;60: 106–13. Available: http://www.ncbi.nlm.nih.gov/pubmed/10646861 10646861

[31] Velazquez MA, Spicer LJ, Wathes DC. The role of endocrine insulin-like growth factor-I (IGF-I) in female bovine reproduction. Domest Anim Endocrinol. 2008;35: 325–42. 10.1016/j.domaniend.2008.07.002 18703307

[32] Davis ME, Boyles SL, Moeller SJ, Simmen RCM. Genetic parameter estimates for serum insulin-like growth factor-I concentration and ultrasound measurements of backfat thickness and longissimus muscle area in Angus beef cattle. J Anim Sci. 2003;81: 2164–70. Available: http://www.ncbi.nlm.nih.gov/pubmed/12968690 12968690 10.2527/2003.8192164x

[33] Davis ME, Simmen RC. Genetic parameter estimates for serum insulin-like growth factor I concentration and performance traits in Angus beef cattle. J Anim Sci. 1997;75: 317–24. Available: http://www.ncbi.nlm.nih.gov/pubmed/9051453 9051453 10.2527/1997.752317x

[34] Nishimura S, Watanabe T, Mizoshita K, Tatsuda K, Fujita T, Watanabe N, et al Genome-wide association study identified three major QTL for carcass weight including the PLAG1-CHCHD7 QTN for stature in Japanese Black cattle. BMC Genet. 2012;13: 40 10.1186/1471-2156-13-40 22607022 PMC3403917

[35] Lindholm-Perry a K, Kuehn L a, Smith TPL, Ferrell CL, Jenkins TG, Freetly HC, et al A region on BTA14 that includes the positional candidate genes LYPLA1, XKR4 and TMEM68 is associated with feed intake and growth phenotypes in cattle(1). Anim Genet. 2012;43: 216–9. 10.1111/j.1365-2052.2011.02232.x 22404358

[36] Bolormaa S, Porto Neto LR, Zhang YD, Bunch RJ, Harrison BE, Goddard ME, et al A genome-wide association study of meat and carcass traits in australian cattle. J Anim Sci. 2011;89: 2297–2309. 10.2527/jas.2010-3138 21421834

[37] Porto Neto LR, Bunch RJ, Harrison BE, Barendse W. Variation in the XKR4 gene was significantly associated with subcutaneous rump fat thickness in indicine and composite cattle. Anim Genet. 2012;43: 785–789. 10.1111/j.1365-2052.2012.02330.x 22497494

[38] Fortes M, Reverter A. genes associated with testicular development, sperm quality, and hormone levels of inhibin, luteinizing hormone, and insulin-like growth factor 1 in Brahman bulls. Biol …. 2012; Available: http://www.biolreprod.org/content/87/3/58.short 10.1095/biolreprod.112.101089 22811567

[39] Taylor J a, Goubillon M-L, Broad KD, Robinson JE. Steroid control of gonadotropin-releasing hormone secretion: associated changes in pro-opiomelanocortin and preproenkephalin messenger RNA expression in the ovine hypothalamus. Biol Reprod. 2007;76: 524–531. 10.1095/biolreprod.106.055533 17151352

[40] Rosie R, Thomson E, Blum M, Roberts JL, Fink G. Oestrogen positive feedback reduces arcuate proopiomelanocortin messenger ribonucleic Acid. J Neuroendocrinol. 1992;4: 625–30. 10.1111/j.1365-2826.1992.tb00212.x 21554648

[41] Bragdon B, Moseychuk O, Saldanha S, King D, Julian J, Nohe A. Bone morphogenetic proteins: a critical review. Cell Signal. 2011;23: 609–20. 10.1016/j.cellsig.2010.10.003 20959140

[42] Adoligbe C, Zan L, Farougou S, Wang H, Ujjan JA. Bovine GDF10 gene polymorphism analysis and its association with body measurement traits in Chinese indigenous cattle. Mol Biol Rep. 2012;39: 4067–75. 10.1007/s11033-011-1188-1 21805344 PMC3294207

[43] McPherron AC. METABOLIC FUNCTIONS OF MYOSTATIN AND GDF11. Immunol Endocr Metab Agents Med Chem. NIH Public Access; 2010;10: 217–231. 10.2174/187152210793663810 21197386 PMC3011861

[44] Versteyhe S, Klaproth B, Borup R, Palsgaard J, Jensen M, Gray SG, et al IGF-I, IGF-II, and Insulin Stimulate Different Gene Expression Responses through Binding to the IGF-I Receptor. Front Endocrinol (Lausanne). 2013;4: 98 10.3389/fendo.2013.00098 23950756 PMC3738877

[45] Chen L, Deng C-X. Roles of FGF signaling in skeletal development and human genetic diseases. Front Biosci. 2005;10: 1961–76. Available: http://www.ncbi.nlm.nih.gov/pubmed/15769677 15769677 10.2741/1671

[46] Hartati H, Utsunomiya YT, Sonstegard TS, Garcia JF, Jakaria J, Muladno M. Evidence of Bos javanicus x Bos indicus hybridization and major QTLs for birth weight in Indonesian Peranakan Ongole cattle. BMC Genet. BioMed Central; 2015;16: 75 10.1186/s12863-015-0229-5 26141727 PMC4491226

[47] DeAtley KL, Rincon G, Farber CR, Medrano JF, Luna-Nevarez P, Enns RM, et al Genetic analyses involving microsatellite ETH10 genotypes on bovine chromosome 5 and performance trait measures in Angus- and Brahman-influenced cattle. J Anim Sci. American Society of Animal Science; 2011;89: 2031–41. 10.2527/jas.2010-3293 21357449

[48] Cao J, Wei C, Liu D, Wang H, Wu M, Xie Z, et al DNA methylation Landscape of body size variation in sheep. Sci Rep. 2015;5: 13950 10.1038/srep13950 26472088 PMC4607979

[49] Xue M, Zan L, Wang H. A novel polymorphism of the insulin-like growth factors binding protein-5 (IGFBP-5) gene and its association with body measurement traits in Bos taurus [Internet]. The Indian Journal of Animal Sciences. 2011 Available: http://epubs.icar.org.in/ejournal/index.php/IJAnS/article/view/5462

[50] Eppig JT, Blake JA, Bult CJ, Kadin JA, Richardson JE. The Mouse Genome Database (MGD): facilitating mouse as a model for human biology and disease. Nucleic Acids Res. 2015;43: D726–36. 10.1093/nar/gku967 25348401 PMC4384027

[51] Keady SM, Kenny DA, Ohlendieck K, Doyle S, Keane MG, Waters SM. Proteomic profiling of bovine M. longissimus lumborum from Crossbred Aberdeen Angus and Belgian Blue sired steers varying in genetic merit for carcass weight. J Anim Sci. American Society of Animal Science; 2013;91: 654–65. 10.2527/jas.2012-5850 23307841

[52] Xu ZY, Yang H, Li Y, Xiong YZ, Zuo B. Temporal expression of TnI fast and slow isoforms in biceps femoris and masseter muscle during pig growth. Animal. Cambridge University Press; 2010;4: 1541–6. 10.1017/S1751731110000649 22444701

[53] Bogdanovich S, McNally EM, Khurana TS. Myostatin blockade improves function but not histopathology in a murine model of limb-girdle muscular dystrophy 2C. Muscle Nerve. 2008;37: 308–16. 10.1002/mus.20920 18041051

[54] Rimbault M, Beale HC, Schoenebeck JJ, Hoopes BC, Allen JJ, Kilroy-Glynn P, et al Derived variants at six genes explain nearly half of size reduction in dog breeds. Genome Res. 2013;23: 1985–95. 10.1101/gr.157339.113 24026177 PMC3847769

[55] Lango Allen H, Estrada K, Lettre G, Berndt SI, Weedon MN, Rivadeneira F, et al Hundreds of variants clustered in genomic loci and biological pathways affect human height. Nature. 2010;467: 832–8. 10.1038/nature09410 20881960 PMC2955183

